# Quality of life of patients with head and neck cancer

**DOI:** 10.5935/1808-8694.20130014

**Published:** 2015-10-14

**Authors:** Mário Rodrigues de Melo Filho, Breno Amaral Rocha, Maria Betânia de Oliveira Pires, Emerson Santos Fonseca, Edimilson Martins de Freitas, Hercílio Martelli Junior, Francis Balduíno Guimarães Santos

**Affiliations:** MSc; Professor of dentstry and preceptor of the Otorhinolaryngology Residency Program of the State University of Montes Claros (UNIMONTES). PhD student in Health Sciences - UNIMONTES; Specialist; Professor of Dentstry - State University of Montes Claros; MSc; Professor of Dentstry - State University of Montes Claros; Student; FaPEMIg Scholarship Holder; MSc. Professor of Dentstry - State University of Montes Claros; PhD. Professor of Dentstry - State University of Montes Claros. Professor of the graduate Program in Health Sciences - UNIMONTES); MSc. Professor of Medicine - State University of Montes Claros and Preceptor of the Otorhinolaryngology Residency Program of the State University of Montes Claros - UNIMONTES). State University of Montes Claros - UNIMONTES e Hospital Santa Casa de Montes Claros

**Keywords:** head and neck neoplasms, quality of life, questionnaires

## Abstract

Patients with head and neck cancer have to deal with the impact of treatment on its functional and aesthetic aspects, and its self-report enables improvements in clinical and social support.

**Objective:**

To evaluate the quality of life of patients dealing with squamous cell carcinoma of the head and neck.

**Method:**

A prospective analytical study. Twenty nine patients with mean age of 57 years answered at three stages: onset, middle and end of treatment, the questionnaires: Quality of Life Core Questionnaire - Cancer 30 and the Quality of Life Questionnaire - Head and Neck, the European Organization for Research and Treatment of Cancer. We used the Friedman test at: 0.05.

**Results:**

There were high mean values concerning physical, cognitive, social functions; improvements in general health and social function decline during treatment; and a significant difference in taste and smell (*p* = 0.020), swallowing (*p* = 0.040), cough (*p* = 0.013) and weight loss (*p* = 0.011).

**Conclusion:**

There was a significant reduction in the quality of life for some common symptoms resulting from cancer treatment, which was not seen in the evaluation of the aspects related to physical, cognitive and social functions, and general health.

## INTRODUCTION

In Brazil, for the year of 2012, there are estimates of 518,510 new cases of cancer, 20,280 of them in the mouth and larynx^1^. The term Head and Neck Cancer is defined on anatomical and topographical bases to describe malignant tumors of the upper aerodigestive tract, including the oral cavity, pharynx and larynx. The most common histological type is the squamous cell carcinoma, present in more than 90% of the cases^2^^,^^3^. Smoking and drinking, when together, have a synergic effect, increasing in 30 fold the risk of the patient developing this type of cancer^3^.

Patients with head and neck cancer, besides harboring a disease which threatens their lives, have to deal with the impact of treatment on functional and aesthetic aspects^4^. This region is the anatomical site of basic functions, such as speech, swallowing, hearing, breathing, associated with social interaction, which are of vital importance to the individual^4^, ^5^, ^6^.

Treatment may be carried out by means of surgery, almost always associated with radiotherapy. Chemotherapy and immunotherapy are relevant adjuvant therapies^7^^,^^8^. Surgery may cause permanent mutilations, loss of organs and/or changes to their functions. Radiotherapy, with or without chemotherapy may cause transient side effects, which subside at the end of treatment; nonetheless, very much limiting to the patient^7^^,^^9^, ^10^, ^11^. Changes to the appearance, voice and difficulties swallowing, when present, bring varied degrees of limitations^4^^,^^9^, ^10^, ^11^, ^12^, ^13^, ^14^, ^15^. Local pain, dyspnea, often times followed by a yellowish, thick and smelly secretion, intermittent cough, chronic fatigue, changes to olfaction, stress, depression and difficulties accepting one's body image; all play in the patient's loss of self-esteem and social isolation. This, and other factors associated with complications, such as mucositis, xerostomia, changes to the sense of taste and infections stemming from the cancer therapy, may all trigger a negative impact on the quality of life of these individuals^4^^,^^14^, ^15^, ^16^, ^17^, ^18^, ^19^, ^20^, ^21^, ^22^, ^23^.

Quality of life is the way with which the individual faces the different aspects of his/her life as a whole. It is associated with the individual's degree of satisfaction found in family life, love life, social and environmental life, and the very existential sense^1^^,^^3^^,^^4^^,^^15^^,^^24^^,^^25^. To assess the quality of life of the patients affected by malignant neoplasia is important to better understand the impact of the disease and its treatment in the patient's daily routine, and improve the care protocol with more encompassing clinical, social and rehabilitation support measures^8^^,^^24^.

The goal of the present study was to assess the quality of life of patients with squamous cell carcinoma in the head and neck, submitted to oncologic treatment.

## METHOD

Analytical, prospective study, carried out in three stages of the antineoplastic treatment: onset, middle and final. We included patients with a histopathological diagnosis of squamous cell carcinoma only in a primary lesion of the head and neck; seen between July of 2010 and June of 2012; with ages equal to or greater than 40 years; who were cognitively able to understand and answer the questions and who had not received previous treatment for this lesion.

In order to assess the patient's cognitive capacity, we employed the *Mini Mental State Examination*(MMSE)^26^ questionnaire. We took off the patients with any type of malignant neoplasia, age below 40 years and who did not have cognitive capacity and were able to communicate.

The patients who accepted to participate in the study and signed the Informed Consent Form, were referred to a dentist who had been trained to collect the data. The patients answered the questionnaires on the day of the first visit with the dentist and established, then, the second endpoint for the second deployment of the questionnaire. The third endpoint was the end of the cancer treatment, when the patient was discharged. Data regarding the disease was collected from the hospital medical chart. The patient answered the two questionnaires at the same time: the EORTC Quality of life questionnaire (QLQ-C 30)^27^ and the Quality of Life Questionnaire-Head and Neck (QLQ-H&N35)^28^ from the European Organization for the Research and Treatment of Cancer - EORTC group, and authorized its use for this study, they were validated in Brazil^24^. The authors advocated the application of the two instruments, which are complementary. The QLQ-C30 encompasses 30 questions grouped into five functional scales, nine scales associated with symptoms and a global scale. The QLQ-H&N35 has 35 questions − 30 which are grouped in 13 scales and five of simple answer. It approaches symptoms associated with the specific tumor location, side effects associated with the treatment provided and additional quality of life aspects affected by the disease or its treatment.

The answers were converted into a linear scoring scale, with values between 0 and 100, as per advocated by EORTC^27^^,^^28^. The results were expressed in mean values, with their respective confidence intervals. A high score in the questions associated with the symptoms reflects their more intense presence, while a high score in the questions associated with function reflects the better life condition of the patient.

The dentist followed the patient throughout treatment, and adjusted the date of the second data collection when treatment was delayed. The participants of this study received dental treatment prior to the cancer treatment, treatment of the oral lesions stemming from the radio and/ or chemotherapy and prosthesis rehabilitation treatment after the cancer treatment, when indicated.

We used the Statistical Package for the Social Sciences, SPSS software, version 17 and we used the Friedman's test for paired samples, with a 0.05 significance level.

The study followed the ethical recommendations for studies with human beings, established in Resolution # 196/96 from the National Health Council, having been approved by means of Ordinance # 2187/10 from the Ethics in Research Committee. There were no risks for the individuals involved in the study, guaranteeing confidentiality and secrecy concerning the information provided by the participants.

## RESULTS

In the study we had 29 patients with ages between 43 and 74, mean age of 57 years, between 2010 and 2012. Most of the patients were men (82.8%), married (75.9%), brown in color (44.8%), coming from other cities of Minas Gerais and referred to treatment in Montes Claros (82.7%), with low schooling (69.0%), rural workers (31.0%), low income, exposed to at least one risk factor (95.5%), almost all were smokers and alcohol drinkers (63.5%).

Tumor size was assessed according to the TNM classification^29^, and T3 and T4 corresponded to 45.2% of the cases. The primary lesion was more frequently located on the tongue or pharynx (22.7% each), followed by the larynx (18.2%), lips (13.6%) and palate, ear or vocal folds (4.5% each). Concerning the cancer treatment employed, most of the patients were submitted to radiotherapy only (44.5%), followed by combined treatment with chemo and radiotherapy (33.3%) and surgery and radiotherapy (18.5%), besides surgery, radiotherapy and chemotherapy, combined (Table 1).

**Table 1:** Patient distribution according to socio-economic and demographic characteristics and treatment type. Montes Claros, 2010-2012.

Variable	n	%
Gender		
Male	24	82.8
Female	05	17.2
Age range		
≤ 50 years	04	13.8
51 a 60 years	13	44.8
> 60 years	12	41.4
Skin color		
White	11	37.9
Brown	13	44.8
Black	05	17.2
Marital status		
Single	02	6.9
Married/stable union	22	75.9
Divorcee/separated	04	13.8
Widow/widower	01	3.4
Income*		
No income	01	3.8
< 1 m.w.	07	26.9
1 to 3 m.w.	18	69.2
Years of schooling		
Illiterate/semi-illiterate	10	34.5
≤ 4 years	10	34.5
5 a 8 years	06	20.7
9 a 12 years	02	6.9
> 12 years	01	3.4
Occupation		
Public servant	01	3.4
Worker in the private sector	02	6.9
Autonomous professional	03	10.3
Domestic worker	01	3.4
Rural worker	09	31.0
General service	02	6.9
Retiree	08	27.6
Without occupation	03	10.3
Treatment type*		
Surgery + Radiotherapy	04	18.5
Chemotherapy + Radiotherapy	09	33.3
Surgery + Chemotherapy + Radiotherapy	01	3.7
Radiotherapy alone	12	44.5
Total	29	100.0

* Three patients without information; n total = 29 patients; n = absolute frequency; % = relative frequency. m.w.= minimum wage

Table 2 shows the mean scores of functions and symptoms for general quality of life, the QLQ-C30, comparing the three prospective cohorts. There were no statistically significant differences among the three moments for any of the functions or symptoms. We see that high mean scores for the patient's functions suggest, in a general view, low disease impact on the physical, emotional, cognitive and social functions, and personal role performance. There was an improvement in the general health status and decline of the social function throughout the treatment. The personal role performance was lower in the middle of treatment and the emotional function was assigned the lowest mean value among all the functions. Considering the scale of symptoms, the occurrence of insomnia was higher at treatment onset; however, nausea, vomit, pain, loss of appetite and constipation were higher in the middle of the treatment.

**Table 2:** EORTC QLQ C30 of patients with cancer assessed at the onset, middle and end of treatment. Montes Claros, 2010-2012.

Items of scale	Treatment period			*p*-value*
	Onset	Middle	Post	
	Mean (SD)	Mean (SD)	Mean (SD)	
Functions				
Physical function	81.6 (19.7)	83.3 (25.3)	83.3 (25.3)	0.751
Emotional function	76.5 (29.8)	84.3 (13.5)	77.9 (21.6)	0.502
Cognitive function	91.7 (18.3)	95.8 (7.4)	91.7 (17.2)	0.861
Social function	91.2 (15.7)	90.2 (15.7)	81.4 (27.6)	0.291
Role performance	86.3 (16.9)	75.5 (22.9)	82.4 (29.1)	0.079
Symptoms				
Fatigue	17.0 (23.3)	13.1 (18.9)	18.3 (30.2)	0.926
Nausea and vomits	4.9 (11.4)	12.7 (24.7)	6.9 (20.5)	0.200
Pain	21.6 (29.3)	23.5 (26.4)	17.6 (19.9)	0.979
Dyspnea	6.3 (18.1)	12.5 (26.9)	18.8 (32.1)	0.368
Insomnia	27.5 (35.8)	7.8 (14.6)	13.7 (29.0)	0.182
Loss of appetite	13.7 (29.0)	25.5 (38.2)	19.6 (39.2)	0.710
Constipation	2.0 (8.0)	19.6 (35.7)	11.8 (28.7)	0.180
Diarrhea	1.9 (8.1)	1.9 (8.1)	9.8 (25.7)	0.368
Financial difficulty	25.5 (36.4)	19.6 (29.0)	33.3 (40.8)	0.168
General health status /QoL	66.7 (23.0)	70.1 (16.7)	71.7 (20.4)	0.280

* Friedman's test for paired samples, with a 0.05 significance level; SD: standard deviation; *p*-value = descriptive level of the Friedman's test; n total = 29 patients.

At the end of treatment, we found a higher rate of fatigue, dyspnea, diarrhea and financial difficulties.

The QLQ-H&N35 specific questionnaire, in details on Table 3, shows a difference between the three moments during treatment, highly significant for taste and smell (*p* = 0.020), deglutition (*p* = 0.040), cough (*p* = 0.013), and weight loss (*p* = 0.011). Difficulty of interaction happened especially at treatment onset, with a mean value of 11.6. Pain and swallowing, dental problems, cough and the use of analgesics - were symptoms of higher impact in the middle of treatment. At the end of treatment, the use of food supplements, difficulties to open one's mouth, greater changes in senses (olfaction and taste), speech disorders, social difficulties in feeding, loss in sex drive and a feeling of being sick, had the higher mean values in relation to the other two moments during treatment.

**Table 3:** QLQ-H&N35 of patients assessed with cancer at the onset, middle and end of the treatment. Montes Claros, 2010-2012.

Scale items Symptoms	Treatment period			*p*-value*
	Onset	Middle	After	
	Mean (SD)	Mean (SD)	Mean (SD)	
Pain	15.6 (20.0)	23.4 (20.0)	19.8 (21.5)	0.288
Senses (taste and smell	11.5 (20.8)	32.3 (33.0)	36.5 (39.5)	0.020
Swallowing	12.3 (23.8)	24.5 (29.4)	21.1 (30.6)	0.040
Problems with speech	12.5 (17.2)	21.5 (26.4)	22.2 (28.4)	0.756
Social/feed problems	7.4 (14.1)	18.1 (25.7)	20.6 (29.3)	0.239
Interact difficulties	11.6 (19.1)	5.8 (7.5)	8.4 (13.9)	0.836
Sexuality	20.0 (27.6)	30.0 (34.6)	31.1 (42.7)	0.236
Problems w/ teeth	27.5 (39.5)	37.3 (48.4)	33.3 (42.9)	0.682
Difficulty opening mouth	19.6 (35.5)	29.4 (37.0)	33.3 (37.3)	0.209
Dry mouth	21.5 (20.2)	31.4 (29.9)	31.4 (39.9)	0.689
Sticky saliva	21.6 (26.2)	29.4 (37.0)	29.4 (35.1)	0.717
Cough	9.8 (15.6)	58.8 (67.7)	37.2 (35.1)	0.013
Feels sick	15.7 (20.8)	15.7 (23.9)	19.6 (33.4)	0.975
Takes analgesic	41.2 (50.7)	52.9 (51.4)	35.3 (49.2)	0.529
Food supplement	5.9 (24.1)	5.9 (24.2)	23.5 (43.7)	0.165
Feeding with a probe	5.9 (24.3)	11.8 (33.2)	11.8 (32.2)	0.779
Weight loss	29.4 (46.9)	64.7 (49.3)	64.7 (49.2)	0.011
Weight gain	23.5 (43.7)	23.5 (43.5)	47.1 (51.4)	0.202

* Friedman's test for paired samples at a significance level of 0.05; SD= standard deviation; p-value = descriptive level of the Friedman's test; n total = 29 patients.

## DISCUSSION

The sociodemographic and economical aspects of the participants in this investigation are in agreement with the epidemiological profile of head and neck cancer found in the literature, men older than 45 years, brown skin, rural workers exposed to at least one risk factor, such as exposure to sunlight, smoking or alcohol, with low economical situation^1^.

Of all the patients assessed, 44.5% were eligible to radiotherapy only, and 33-3% were treated with radiotherapy associated with chemotherapy. Technological progresses concerning radiotherapy procedures and the new chemotherapy protocols have favored the choice of theses means of treatment. Nonetheless, there is the risk of increasing toxicity induced by chemo-radiation with painful and debilitating effects, which can cause the need to interrupt treatment, compromising the diagnosis of the patient^3^^,^^6^^,^^30^. Different treatment modalities for some tumor sites in the head and neck may bring about survival results and similar disease control, however, with different complications, sequelae and functional results. These induced effects and sequelae may negatively influence the quality of life of individuals which could, in the future, help in clinical judgment and the definition of the treatment approaches. In this context, the interest for the quality of life of these patients is directly associated with the day-to-day care practices in health centers^3^^,^^7^^,^^14^^,^^15^^,^^18^^,^^23^^,^^25^^,^^31^^,^^32^.

There were no statistically significant differences between the three moments for no function or symptom in the QLQ-C30 questionnaire assessment (Table 2). The mean scores of the functions assessed were high in the treatment onset, and changed very little prospectively. In a similar fashion, the global quality of life did not suffer any significant change. Similar results were found by Braam et al.^6^ in the assessment of 44 patients submitted to neck-facial radiotherapy only, or adjuvant to surgery. Notwithstanding, Bansal et al.^30^, assessed 45 patients with indications for head and neck radiotherapy, and showed a correlation between the worsening of the physical function and an increase in symptoms such as: fatigue, pain, loss of appetite. Blanco et al.^12^ showed an increase in the symptoms scale (pain, fatigue and weight loss) and decline on the functional scale, with loss of physical, social and emotional functions and role performance. In the present study, except for pain and insomnia, none of the symptoms returned to their baseline values upon treatment discharge.

We found a low disease impact on their physical, emotional, cognitive and social functions and personal role performance, different from Scharloo et al.^33^, in a prospective study involving 177 patients, in which there was an improvement in the emotional function and a worsening in social function throughout the follow up period. The patients from Montes Claros had the lowest mean value for the emotional function among all the functions; nonetheless, there was a large data variation prospectively, which may reflect the large subjectivity of these parameters. Moreover, there was a drop in the social function throughout treatment. In one study carried out by Connor et al.^4^, the patients had a progressive worsening of their physical function, contrasting with this study, in which there was an improvement in this functional scale throughout the period, and an improvement in their general health status.

Social interaction was more impaired at the onset of the anti-cancer treatment and improved in the middle of the treatment (Table 3). Opposite results were found by Ohrn et al.^34^ using the same questionnaires in the assessment of 18 patients. According to the authors, feeding and social contact had higher intra and post-radiotherapy mean values.

Comparing mean values at the end of treatment to the values found at treatment onset and middle of the treatment in the QLQ-H&N35 questionnaire assessment (Table 3), there is an increase in dry mouth, saliva viscosity with a significant swallowing impairment (*p* = 0.040). This can be partly associated to the surgical procedures utilized in the treatment of head and neck tumors^5^, although the number of patients operated in this sample is small. Other possible factors participating are the greater presence of primary tumors in aerodigestive sites, such as the tongue and pharynx (22.7% each), and non-surgical treatment, such as chemo-radiotherapy, in organ preservation pro-tocols^5^. The increase in nausea and vomit symptoms, loss of appetite and constipation seen during the middle of the treatment may be associated with the appearance of the classic radio and chemo-induced acute affects.

Another common complication is dysgeusia, distorted or impaired sense of taste, affecting 50% to 75% of those submitted to radiotherapy, chemotherapy, or both^14^^,^^35^. There was a reduction in taste and olfaction in the three periods assessed (11.5-32.3-36.5, respectively, with a *p* = 0.020). It is believed that this worsening in taste happens with the cumulative dose of radiation^14^^,^^35^. About 15% of these individuals continue to have dysgeusia, even after the end of treatment^14^^,^^35^, ^36^, ^37^. These symptoms may have contributed to the progressive weight loss reported by the patients (*p* = 0.011).

A proper nutrition is important and needs to be stimulated and facilitated^35^, ^36^, ^37^, despite the increase in using food supplements at the end of the oncologic treatment (Table 3). Changes to olfaction and taste associated with the difficulty in opening ones mouth and feeding-related social difficulties may have contributed to the increase in using food supplements. Blanco et al.^12^ found a greater occurrence of changes in the sense, especially after 3 months of the surgery; however, with partial improvement after 6 months.

In our study, pain was present in the three months of the assessment, with similar responses in the two questionnaires. It is predominant in the middle of the treatment, and it declines at its end. The high standard deviation from the mean suggests a large variability in its perception. The pain increase during treatment may be due to adverse opportunistic lesions arising from the radiotherapy and/or the chemotherapy, which may justify the increase in analgesic medication use (Table 3). Pain is common in patients with head and neck cancer. It is reported by about half of the patients before cancer treatment, by 81% of them during treatment, by 70% at the end and by 36% six months or more after treatment^38^. It may be a consequence of the curative treatment associated with the malignancy, to physical and psychological suffering, and because of nociceptive and neuropathic mechanisms. In some cases, pain is a coincidence which is not directly associated with the cancer^38^.

To assess the quality of life of cancer patients is complex, considering the large number of variables which impact the patient's self-perception, from their social situation all the way to the very particularities of their diseases. It encompasses individual assessment characteristics, which does not depend on the patient's system of beliefs, values and even physical strength^3^^,^^5^^,^^15^. For these reasons, it is a fundamental tool used to assess the impact of the disease and its treatment obtaining epidemiological evidence which support changes to a more effective multiprofissional support protocol for the patients^5^^,^^8^^,^^17^^,^^19^^,^^24^.

## CONCLUSION

There was a significant quality of life reduction for the patients throughout treatment in relation to some common symptoms in the treatment of cancer, which did not occur in the assessment of the aspects associated with the social, cognitive and physical functions. It is necessary that the multidisciplinary team use information obtained in this investigation in order to build a broader care protocol, involving demands arising from symptoms and life situations.
